# Prosthetic Knee Joint Infection due to Salmonella Species: A Case Report

**DOI:** 10.7759/cureus.8519

**Published:** 2020-06-09

**Authors:** Ali Ghaffar, Malik Hatim Hussain, Rama Mohan

**Affiliations:** 1 Orthopaedics and Trauma, East Lancashire NHS Hospitals, Blackburn, GBR; 2 Trauma and Orthopaedics, North Manchester Hospital, Manchester, GBR

**Keywords:** septic arthritis, prosthetic joint infection, salmonella

## Abstract

We present a case of a 79-year-old male with a Salmonella enteritidis prosthetic knee joint infection preceded by an episode of profuse diarrhea. The infection was treated with ceftriaxone antibacterial chemotherapy, and an arthroscopic knee joint washout. The initial treatment failed to eradicate the Salmonella infection. A second open washout procedure with the replacement of knee joint insert was performed along with the addition of ciprofloxacin to the ongoing ceftriaxone which eventually eradicated the infection. Although S. enteritidis is a very rare cause of prosthetic knee infection, suspicion of Salmonella as a potential causative agent should be borne in mind particularly if the onset of the symptoms is preceded by gastrointestinal manifestations.

## Introduction

Besides improving the quality of life, joint replacements are considered as life-enhancing surgical procedures [1]. About 719000 knee replacements were performed in the United States in 2010 alone [2]. According to previous reports, 1%-2% of joint replacements eventually become infected [3]. Prosthetic joint infection/periprosthetic joint infection involves prosthesis and adjacent tissues [1]. While Gram-positive Staphylococcus is a common culprit, enteroinvasive Gram-negative organisms (Salmonella) can rarely infect replacement prosthesis, especially in immunocompromised individuals [4-5]. We report a case of a prosthetic joint infection caused by *Salmonella enteritidis* in an immunocompetent male.

## Case presentation

A 79-year-old male presented with pain and swollen left knee. He was febrile and unable to weight bear on the left knee joint. There was no history of trauma. He had recently returned from a holiday in Cuba a few days prior to the presentation. Whilst in Cuba, he developed profuse diarrhea which spontaneously resolved within three days. This was soon followed by acute onset of mild, progressively worsening, left knee pain. 

His past medical history was significant for atrial fibrillation, mild aortic stenosis, benign prostatic hyperplasia, and bilateral total knee arthroplasty. A cruciate retaining left total knee arthroplasty was performed in 2004. Postoperative recovery was satisfactory and normal limb function was achieved.

At the time of his current admission, the temperature was 38.6°C. Initial blood investigations showed a white cell count (WCC) of 14.5 x 109/L and C-reactive protein (CRP) 229 mg/dL. On examination, moderate amount of effusion was noted in the left knee joint along with erythema over the joint. Range of movement in the left knee was restricted, systemic examination was unremarkable. Aspiration fluid was turbid and cultures grew *S. enteritidis* which was sensitive to ceftriaxone and ciprofloxacin. The patient was commenced on ceftriaxone (2 g/24 hours IV) and fasted in preparation for arthroscopic knee washout on the following day.

An arthroscopic washout was performed and approximately 150 mL of pus was drained. Following this, the CRP showed mild degree of improvement, meanwhile WCC increased to 16. 5 x 109/L on the day 3 of the washout. Clinically the knee remained painful and swollen, and the temperature was 37.3°C. Ceftriaxone therapy was continued throughout this time. Poor response to the initial washout led to a decision to perform an open surgical washout with polyethylene insert exchange. Following this, both the CRP and WCC showed drastic improvements. As per recommendations by the infectious diseases team, ciprofloxacin (750 mg BD, PO) was also added to the existing IV ceftriaxone therapy to ensure complete extermination of *S. enteritidis*. On day 13 following the washout, WCC was 9.4 x 109/L and CRP 97 mg/dL. The patient was pain free and apyrexic. Joint swelling had disappeared. The patient was subsequently discharged.

The patient was in good clinical condition at the three-month follow-up. The laboratory findings and knee examination were all normal with a pain-free range of movement of 0°-110°.

## Discussion

Prosthetic joint infection is a rare phenomenon. It has been previously reported that only about 1%-2% of all cases involving a prosthetic joint become infected. Staphylococcus is responsible for the majority of the cases [3]. Conditions such as rheumatoid arthritis, systemic lupus erythematosus (SLE), sickle cell disease, and HIV appear to be associated with a relatively higher frequency of Salmonella bacteremia and septic arthritis [6].

Salmonella is contracted through fecal-oral route. Gastrointestinal complications are more common manifestation due to *S. enteritidis* infection [7].

In the present case, there were no indications that the patient was immunocompromised, either iatrogenically or as a result of a medical condition. Focal Salmonella infection of the left knee joint is likely to have occurred due to occult bacteremia during the preceding episode of diarrhea.

Our patient had successful outcomes from a combination of antibacterial therapy and surgical intervention that also involved polyethylene insert exchange. The need to fortify antibacterial therapy with a combination of two drugs raises concerns of drug resistance in *S. enteritidis*.

## Conclusions

In conclusion, although *S. enteritidis* is a very rare cause of prosthetic joint infection, the possibility of prosthetic joint infection caused by *S. enteritidis* should be borne in mind especially when patients present with history of gastrointestinal complications or recent travel.
